# Analysis of Factors Influencing Drug Prices Differently in Official Drug Pricing and Cost-Effectiveness Evaluation

**DOI:** 10.3390/jmahp14030053

**Published:** 2026-08-31

**Authors:** Mariko Hara, Hiroshi Nakamura

**Affiliations:** Graduate School of Business Administration, Keio University, Kanagawa 223-8526, Japan; nakamura.z3@keio.jp

**Keywords:** Japan, cost-effectiveness evaluation, drug pricing, health technology assessment, pricing premium

## Abstract

Background: In Japan, official prices of pharmaceuticals have been determined by efficacy and safety. In 2019, Cost-Effectiveness Evaluation (CEE) was introduced to adjust these prices according to Incremental Cost-Effectiveness Ratios (ICERs). Objectives: To analyze the differences in the factors influencing drug price between the initial official drug pricing and subsequent price adjustments based on CEE. Methods: Twenty-three drugs evaluated in Japan between 2019 and May 2025 were descriptively analyzed using public reports on official drug pricing and CEE. Results: Four differences were identified: (1) Official initial drug pricing grants premiums for certain characteristics that are not directly associated with improvements in Health-Related Quality of Life (HRQoL), whereas such premiums are not considered in CEE. (2) Official drug pricing limits comparators to existing drugs, whereas CEE did not impose such restrictions. (3) Official drug pricing excludes older drugs as comparators to reward innovation, whereas they were not excluded in CEE. (4) CEE requires sufficient data for CE analysis, such as clinical trial or real-world data, for comparators, unlike official drug pricing. Factors (1) and (3) were most frequent. Conclusion: Reducing these differences and maintaining consistent innovation evaluations may enhance price predictability and support sustainable pharmaceutical investments in the Japanese market.

## 1. Introduction

In Japan, changes in medical technologies and demographic shifts such as declining birthrate and an aging population have increasingly highlighted the critical need to balance public health insurance system with the evaluation of innovation [1]. Within this context, the Japanese official drug pricing system has long contributed to the broad and prompt inclusion of pharmaceuticals under public health insurance, thereby facilitating smooth access to medical care. After regulatory approval, an official reimbursement price is determined, and the product becomes reimbursable within 60–90 days [2].

Official drug prices are calculated based on two primary pricing methods. When comparable drugs exist in the market, a similar-efficacy comparison method is applied, in which prices are set based on the prices of existing drugs. Comparable drugs are identified by their indications, mechanisms of action, chemical structures, and routes or forms of administration.

When no comparable drugs exist in the market, a cost accounting method is applied, in which prices are calculated based on the manufacturing costs and other related expenses of the newly developed product [3].

In both methods, premiums are granted for various reasons, including mechanism of action, efficacy, safety, convenience, market rarity, and accelerated market entry, if the requirements for premium are fulfilled. Since 2014, these premiums related to utility have been converted into point-based evaluations to determine corresponding premium rates [4,5] (see Appendix A Table A1 for details on each point). There are four categories for premium;

(i) Novel Mechanism of action

(ii) Higher efficacy or safety compared to the existing similar drugs (incl. evidence level)

(iii) Improvement treatment in the treatment method of the target disease

(iv) Higher usefulness due to formulation improvements compared to the existing similar drugs

Pricing premiums in the Japanese drug pricing system are granted for attributes that are considered to provide added value over existing treatment options. However, Japan also employs a cost-based pricing method for products that lack comparable drugs. Accordingly, the pricing system incorporates a wide range of premium categories to capture different types of therapeutic and societal value. For example, a premium may be granted to a product that establishes a new standard of care in an area where no standard treatment previously existed and subsequently becomes the standard of care both domestically and internationally. Other premium categories recognize improvements in patient convenience, such as reduced dosing frequency.

The Japanese cost-effectiveness evaluation (CEE) system was introduced in 2019 and it is applied to selected products (see Appendix A Table A2 for selection criteria) that have a substantial impact on the national healthcare budget, typically those meeting specific criteria [6].

While Japan’s CEE system functions as part of the official drug pricing system to adjust drug prices, several European countries use CEE or health technology assessment (HTA) to determine reimbursement eligibility or reimbursement levels [7]. This Japanese CEE primarily targets the premium portion obtained through the initial drug pricing process (i.e., the portion attributed to innovation) for price adjustments, with some exceptions.

Although the methodological approach to cost-effectiveness evaluation in Japan is broadly similar to that used in countries such as the United Kingdom, its role within the healthcare system differs fundamentally. Because CEE is conducted after reimbursement and price listing, its results are used exclusively for post-listing drug price adjustments rather than for reimbursement decision-making, distinguishing the Japanese system from those of other countries.

Compared with Japan, CEE was introduced earlier in many countries, particularly in Europe, where it has been adopted for a variety of policy purposes. Table 1 summarizes the characteristics of CEE systems in selected countries.

The differences between the two systems can be broadly categorized into institutional aspects and operational aspects.

From an institutional perspective, the Japanese CEE system differs from those of other countries in that it is conducted after drug pricing and reimbursement decisions have been made and is applied only to selected products with a substantial financial impact that have received a pricing premium. Furthermore, the results of CEE are used exclusively for post-listing price adjustments, specifically for the premium portion of the drug price. Consequently, the additional value recognized during the initial drug pricing process is effectively assessed twice, raising the question of whether consistent value judgments are obtained across the two evaluation processes.

From an operational perspective, potential inconsistencies may arise in both the value components assessed and the selection of comparator technologies. While the drug pricing system evaluates a wide range of value elements through pricing premiums, CEE primarily relies on the incremental cost-effectiveness ratio (ICER), which may not fully capture certain value attributes, such as convenience and accessibility. In addition, differences in the comparator technologies used for CEE and those adopted during the drug pricing process may lead to further inconsistencies between the two evaluation frameworks. The key differences between the two systems are summarized in Table 2.

Furthermore, the current framework and operational rules do not explicitly consider the linkage between the initial drug pricing process and CEA. As a result, the consistency between these two evaluation processes has not been systematically examined. This lack of alignment represents a key operational issue and constitutes the primary focus of the present study.

Theoretically, the differences between the two systems identified in this study are considered to reflect the historical context in which Japan’s pharmaceutical pricing system has incrementally evolved. As the official drug pricing system operated over many years, it established its own distinct evaluation framework; subsequently, CEE based on ICER was introduced as an overlay onto this existing system. Consequently, despite sharing common policy goals, the two systems have come to operate under divergent evaluative structures. The resulting discrepancies in value assessment and comparator selection can thus be interpreted as an institutional misalignment rooted in path dependence [11].

### Objectives and Related Literature

As noted above, the Japanese CEE system is a component of the official drug pricing system. Both systems have an impact on drugs’ official price; therefore, the objective of this study is to examine whether the two systems were consistent in their evaluation of pharmaceutical value. Notably, no previous research has directly compared Japan’s official drug pricing system with its CEE system.

Previous studies have primarily addressed the related but distinct aspects of CEE or HTA and economic evaluations. For example, Konishi [12] reported that the number of HTA-related publications in PubMed has been increasing in Japan, although the growth rate has remained considerably lower than that in the United States, United Kingdom, and China. Other studies have focused on specific components of cost-effectiveness analyses, such as cost elements and analytical methodologies, as examined by Kashiwa [13], Yanagi [14], Nakagawa [15], and Noda [16]. Fukuda [17] presented comparative analyses of healthcare systems, including the role of CEE and national CEE guidelines. More recently, Hirano [18] investigated differences in analytical components between manufacturers’ submissions and public assessments within the Japanese CEE framework.

## 2. Materials and Methods

### 2.1. Study Design

This study employed a descriptive analytical design to examine pharmaceutical products that underwent the Japanese CEE process.

### 2.2. Data Sources

Data were obtained from publicly available reports on official drug pricing and CEE, published between May 2019 and May 2025. Official CEE reports were retrieved from the Center for Outcomes Research and Economic Evaluation for Health (C2H; Wako, Saitama, Japan) [19]. Official reimbursement price announcements were obtained from the Central Social Insurance Medical Council (Chuikyo) [20].

### 2.3. Selection of Materials

We screened CEE reports and price announcements issued between 2019 and May 2025. Of the products subject to the CEE, 44 had completed the CEE process. Among these, 23 products were included in the present study after excluding 9 products that did not undergo independent analysis as analogous products, 3 medical devices, and an additional 9 products for which no premium was actually granted in the official drug pricing.

Among these, 9 products were priced using the cost-based pricing method. Although they qualified for innovativeness/usefulness premiums, the effective premium rate was reduced to zero under the drug pricing rules because the premium coefficient was lowered due to insufficient disclosure of cost information. For the purposes of this analysis, these products were treated as having no recognized premium and were excluded from the analysis. A summary of the process used to narrow down the products to 23 is shown below (Table 3), and detailed information for each product is provided in Appendix A Table A3.

### 2.4. Data Extraction

For the 23 products, the following key information was extracted:

- Target population;
- Comparators used in both the official drug pricing and the CEE;
- The initial reimbursement price, the official drug pricing method and granted premiums;
- The subsequent price adjustment based on CEE results.

### 2.5. Data Analysis

Elements in official drug pricing and CEE were descriptively analyzed. Factors influencing drug prices differently in official drug pricing and CEE results were identified and categorized. The consolidated data were further analyzed according to the type of price adjustment after CEE. When multiple analysis populations were specified, a product was considered to meet the criterion if it satisfied the criterion in at least one of the analysis populations.

### 2.6. Ethical Considerations

This study involved the analysis of publicly available aggregate data obtained from official government reports. No individual patient-level data were accessed or analyzed. Accordingly, ethical review and approval were not required, and informed consent was not applicable.

## 3. Results

**Figure 1:**
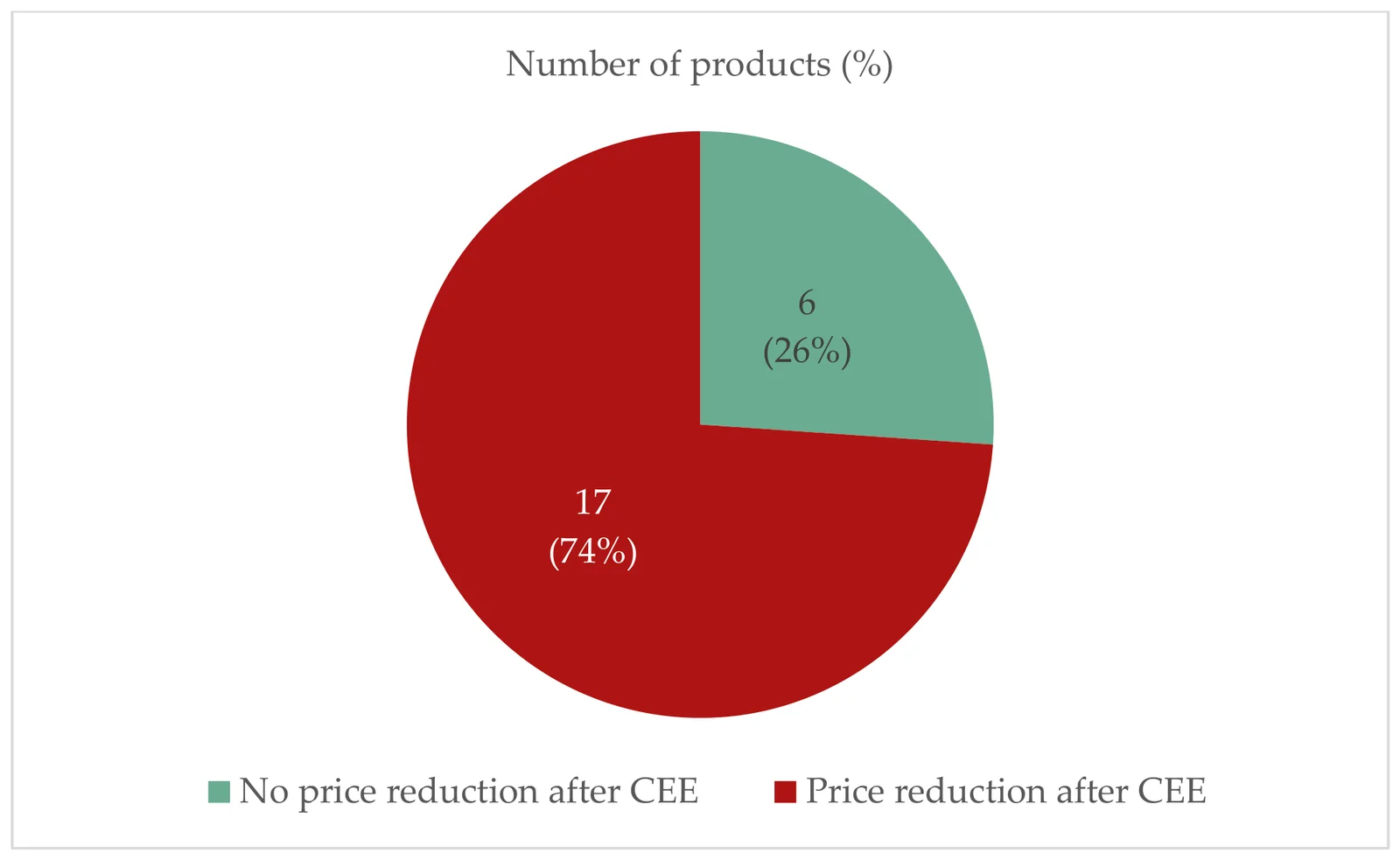
Price reduction after CEE for products granted a premium in official drug pricing (number of products).

Among 23 products that received a premium in official drug pricing, 17 products (74%) experienced price reductions and no product experienced a price increase after CEE (Figure 1).

### 3.1. Factors Driving Differences Between Official Drug Pricing and CEE

The factors influencing these differences in evaluation were classified into the following four categories: (1) In official drug pricing, characteristics not directly related to improvements in health-related quality of life (HRQoL) were also valued, whereas such characteristics were not considered in CEE. (2) In official drug pricing, comparator drugs were limited to existing drugs, whereas CEE did not impose such restrictions. (3) In official drug pricing, older existing drugs were not selected as comparators to reward innovation, whereas they were not excluded in CEE. (4) In CEE, sufficient data for analysis, such as clinical trial or real-world data, were required for treatment to be used as a comparator; however, this was not necessarily required in official drug pricing.

#### 3.1.1. Innovation Not Directly Related to HRQoL Improvement in CEE

**Figure 2:**
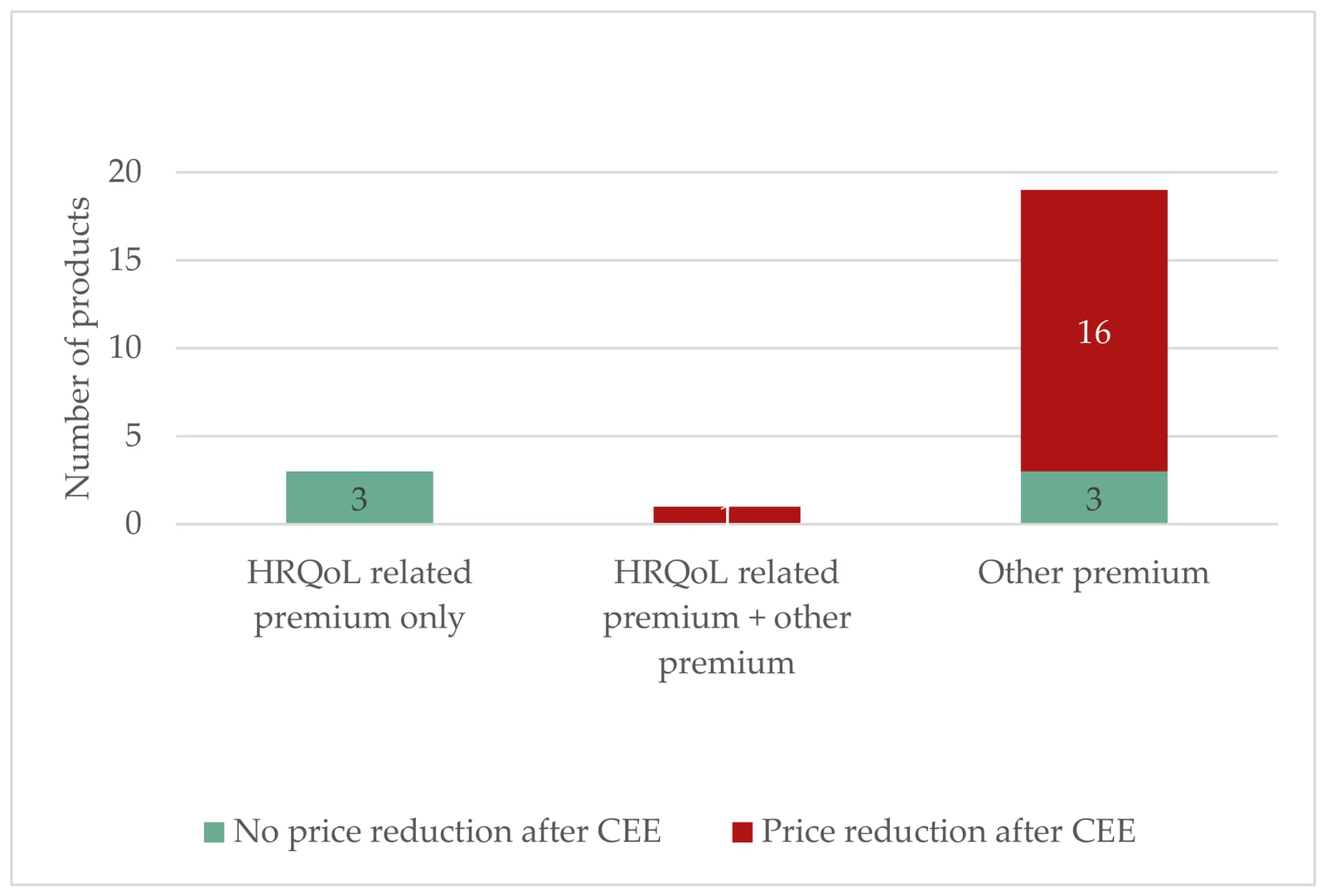
Type of premiums and downward price adjustment after CEE.

Focusing on the type of premiums in official drug pricing, all three products granted only the HRQoL-related premium maintained their prices, whereas many products granted additional premiums experienced price decreases (Figure 2). Premiums related to HRQoL, such as those for efficacy and safety, appear to directly influence quality-of-life outcomes considered in the CEE, whereas other premiums are less likely to be reflected in the evaluation results.

Other premiums were granted to products demonstrating a novel and clinically useful mechanism of action (MOA), evidence of improvement in the treatment of the target disease, or higher clinical usefulness attributable to formulation improvements (Table 4). Specifically, these criteria included a mechanism of action with a site of action or target molecule distinct from those of existing products; improved treatment outcomes in patient populations for whom existing therapies are insufficiently effective or cannot be used because of safety concerns or other reasons, establishment as a standard-of-care treatment, a substantially faster onset of action, a markedly longer duration of efficacy, or significantly greater convenience in use compared with existing treatment modalities; and formulation innovations that provide enhanced clinical utility, such as a marked reduction in the invasiveness associated with drug administration. These aspects are considered difficult to adequately capture within quality adjusted life years (QALYs) measured using instruments such as EQ-5D. As shown in Figure 2, while most products were granted premiums other than the HRQoL-related premium, the majority of these products experienced price reductions following the CEE. In contrast, the prices of all three products granted the HRQoL-related premium were maintained following the CEE.

#### 3.1.2. Older Drugs as Comparators in the Evaluation of Innovation

Differences in comparator selection are common between official drug pricing and CEE. Even when drug prices are calculated based on comparisons with existing drugs using the similar-efficacy comparison method (i.e., an existing drug is used as a comparator in both official drug pricing and CEE), in approximately 90% of CEEs, the comparator drug selected was different from that used for official drug pricing.

The cost-accounting method is applied only when no comparable drug is identified under the official drug pricing. Therefore, drugs priced using the cost-accounting method do not have comparable drugs for pricing purposes. Consequently, even among the three products out of the 23 that were priced using the cost-accounting method, the comparator technologies used in the CEE differed from those used in the official drug pricing (For one product, a non-drug intervention was included among the comparators in the analysis, whereas two products were compared with older-generation drugs).

In the table below, the comparators used were classified into four categories: (a) the same comparator as that used in the drug pricing process, (b) non-drug comparators, (c) new drugs (i.e., drugs launched within the previous 10 years), which are eligible as comparators under the drug pricing system, and (d) old drugs (i.e., drugs launched more than 10 years earlier), which are not eligible as comparators under the drug pricing system.

Notably, for four products (17%), comparators other than drugs were selected in CEE. In the four products analyzed, non-drug interventions such as surgery, procedural interventions, or watchful waiting were also included as comparators. When no similar drug exists in official drug pricing, the cost-accounting method is applied and no explicit comparator is defined. In contrast, CEE requires a uniquely defined comparator, even in the absence of directly comparable drugs.

#### 3.1.3. Comparator Selection in the Absence of Data at the Time of Evaluation

**Figure 3:**
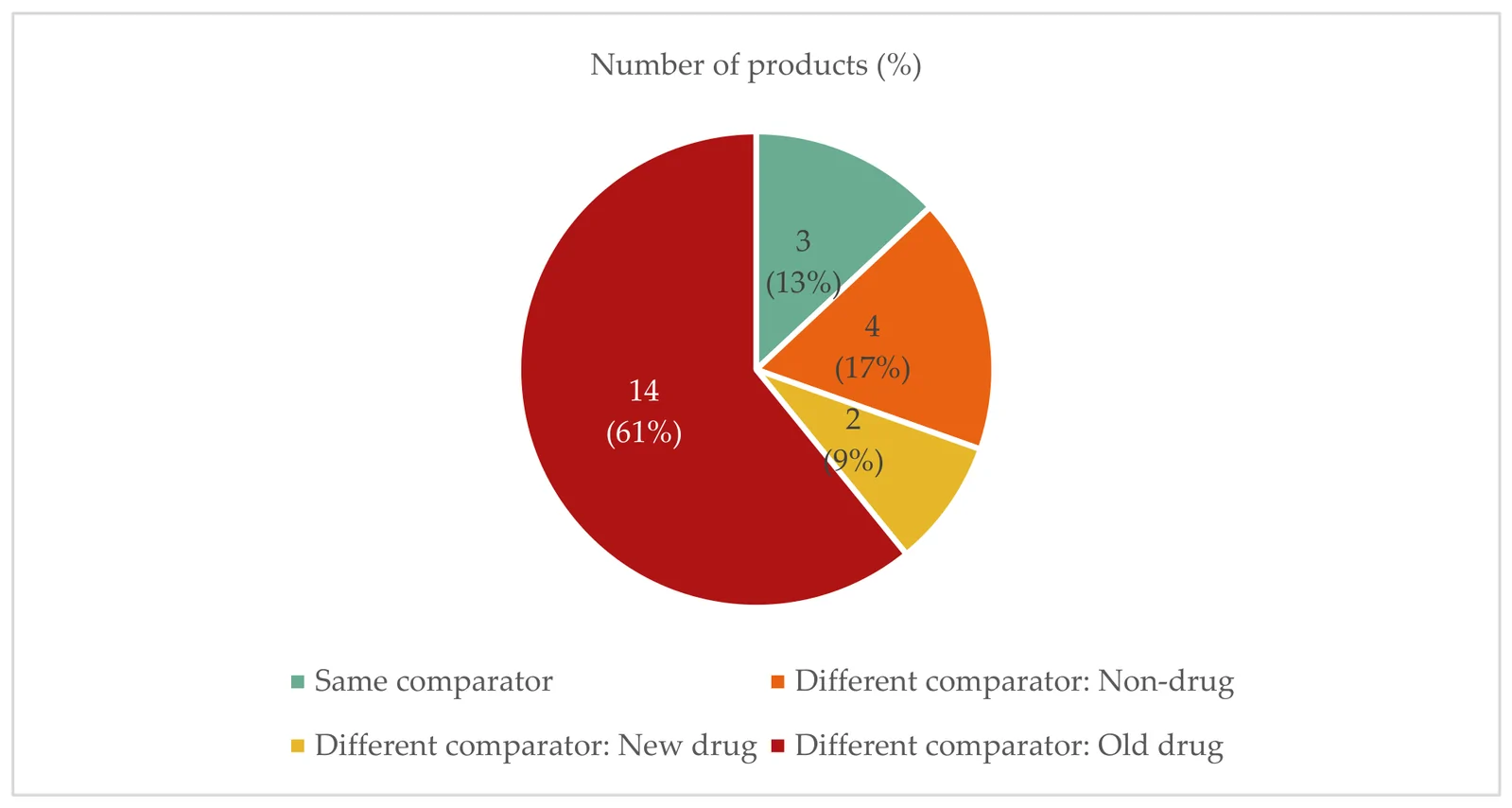
Difference in comparators between official drug pricing and CEE.

There were many cases in which different drugs were used as comparators. Even when drugs were used as comparators, more than half were older drugs that had been marketed for many years, and only three drugs (13%) had the same comparator in both official drug pricing and CEE (Figure 3).

For official drug pricing, older drugs that had been listed in the NHI drug price standard within the previous 10 years and for which no generic equivalent of the comparator drug had been listed are typically excluded as comparators. This system takes into account the fact that drug prices are gradually reduced through price revisions conducted every one to two years. Because the Japanese drug pricing system has no mechanism for price increases, pricing revisions are subject to downward pressure, which may result in prices being reduced beyond levels justified by the clinical value of the drug. Excluding products that have been on the market for more than 10 years from the set of comparators is therefore considered to reflect the assessment of innovation.

In contrast, for CEE, comparators are selected from products that are widely used in clinical practice and are expected to be replaced by new products in the target population. Although criteria for comparator selection are specified in the guidelines, there have been cases in which the comparator selected in practice was the least costly among drugs with similar efficacy, reflecting a greater emphasis on economic evaluation than on novelty [2].

Among the products whose prices were reduced, older drugs tended to be selected as comparators in the CEE (Table 5).

#### 3.1.4. Comparator Selection with Limited or Emerging Evidence

When evidence is limited, comparators are chosen based on the availability of sufficient data rather than demonstrated efficacy or safety in CEE. For new treatment categories with no alternative therapies, comparators are drawn from existing treatments. In some cases, recently launched products may be the most appropriate comparators. However, because the data required for cost-effectiveness analysis are insufficient, these products are not selected as comparators, and instead, “old drugs” are adopted as comparator technologies.

### 3.2. Major Factors

The present analysis revealed several key differences between official drug pricing and CEE.

**Figure 4:**
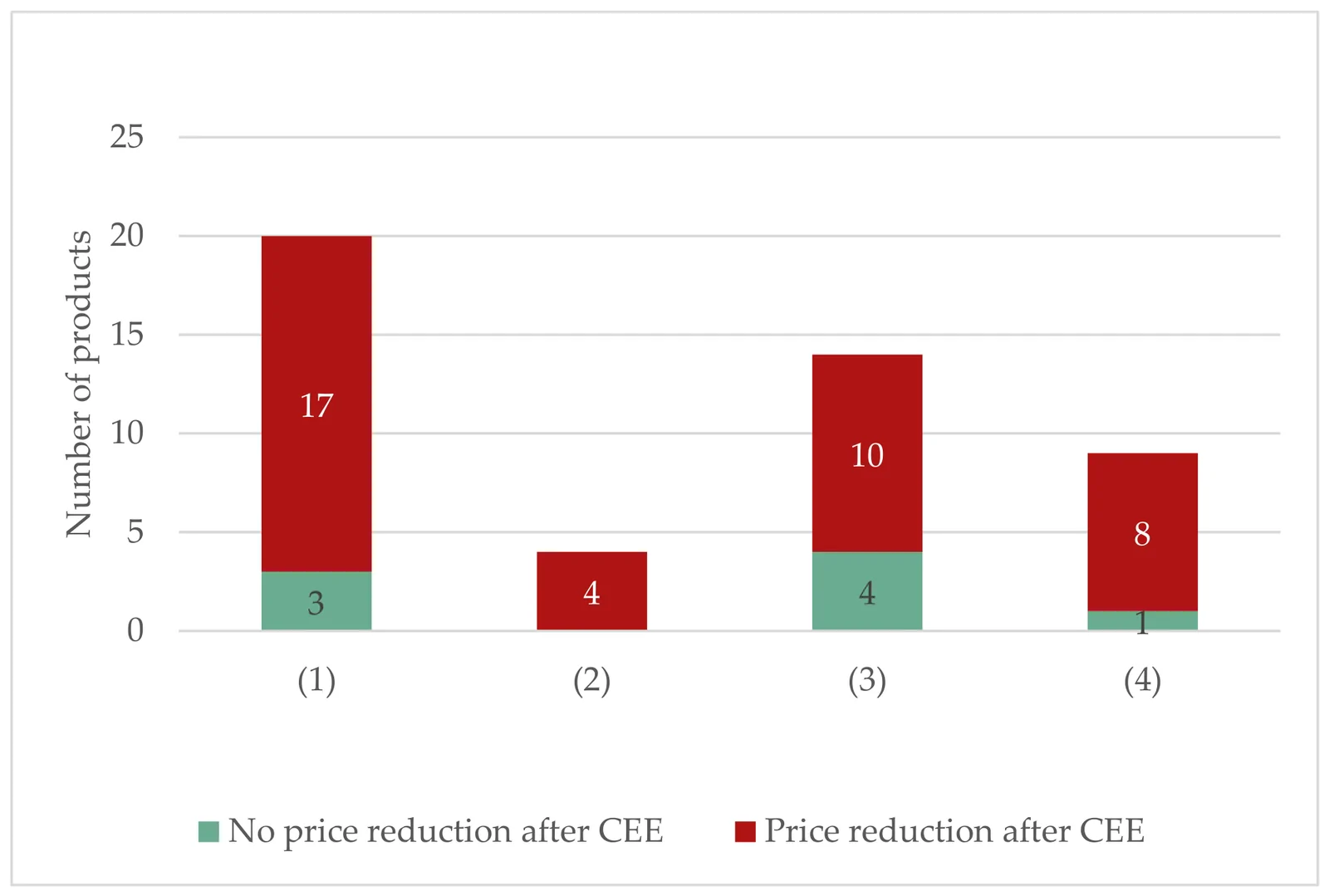
Number of products corresponding to the four major factors (by price adjustment category). Note: (1) In official drug pricing, characteristics not directly related to improvements in health-related quality of life (HRQoL) were also valued, whereas such characteristics were not considered in CEE. (2) In official drug pricing, comparator drugs were limited to existing drugs, whereas CEE did not impose such restrictions. (3) In official drug pricing, older existing drugs were not selected as comparators to reward innovation, whereas they were not excluded in CEE. (4) In CEE, sufficient data for analysis, such as clinical trial or real-world data, were required for treatment to be used as a comparator; however, this was not necessarily required in official drug pricing.

Overall, four major factors were identified as influencing the differences between official drug pricing and CEE (Figure 4). Among these, the most influential factors were (1) Evaluation of innovation that is not directly related to improvements in HRQoL in CEE and (3) older existing drugs were not selected as comparators.

Regarding (1), the CEE framework evaluates value in terms of improvements in HRQoL relative to the comparator technology, whereas the value assessed in official drug pricing encompasses a broader range of elements, including mechanisms of action and improvements in treatment approaches. By limiting the assessment to this narrower scope of innovation, the CEE framework does not fully recognize the premiums granted under the official drug pricing.

Regarding (3), as noted in Section 3.1.4, in official drug pricing, drugs that have been on the market for more than 10 years are generally excluded from the comparator to appropriately reflect innovation. In contrast, such older products may still be included as comparators in the CEE. Consequently, elements of innovation that are comprehensively incorporated into official drug pricing are not fully captured in the CEE.

## 4. Discussion

### 4.1. Sources of Differences Between Drug Pricing and Cost-Effectiveness Evaluation Comparator Selection

First, there is currently no formal process for ensuring alignment between the results of cost-effectiveness evaluation and the drug pricing system. In Japan, no pre-approval consultation process has been established for the selection of comparator technologies for CEE. This is because, before drug listing, it is uncertain which products will ultimately be subject to CEE. A pre-approval consultation process, as implemented in other countries, should be considered.

Second, when no similar drug exists in the drug pricing system, the cost-accounting method is applied and no explicit comparator is defined. In contrast, CEE requires a uniquely defined comparator for each target population, even in the absence of directly comparable drugs. Non-drug interventions such as surgery, procedures, or watchful waiting may serve as comparators in such cases. Even when a comparator exists, an older comparator or one with insufficient data may be chosen in CEE if the newest drug lacks sufficient evidence.

Finally, for official drug pricing, comparators are generally restricted to drugs launched within the past ten years, and elements of innovation are partially considered. In CEE, although the guidelines specify the criteria for comparator selection, the chosen comparator is sometimes the least costly drugs within the same therapeutic category, suggesting that economic value may be prioritized over novelty or similarity of efficacy in comparator selection [21]. Selection may also refer to randomized controlled trial comparators or similar products used in official pricing, and non-treatment can be selected if appropriate [21].

### 4.2. Evaluation of Premiums and Additional Value

First, premiums in official drug pricing recognize various aspects of innovation, including clinical utility, safety, novel mechanisms of action, improvements in convenience, introduction of standard treatments in previously underserved areas, and market size. These premiums are added to the base price [4,5]. In contrast, CEE primarily consolidates additional value in terms of cost and QoL. Certain innovations, such as improvements in treatment methods, including changes in formulation, extended dosing intervals, and enhanced patient convenience, are difficult to capture in QoL measures.

Second, while patient-reported outcomes can theoretically reflect these improvements, instruments such as the EQ-5D are generally insensitive. Recent studies on the EQ-5D 3L and 5L and Japanese-specific conversion tables have highlighted that current utility measures do not fully capture all therapeutic benefits. The HTA Handbook acknowledges these limitations, noting that some elements of treatment innovation cannot be reflected in QoL-based CEE and uniform rules for assessment are difficult to define [22].

Drawing on discussions and experiences from other developed countries, it is necessary to consider the unique feature of the Japanese system, where cost-effectiveness evaluation is used solely for adjusting the premium components incorporated into drug prices.

In the 2026 medical fee revision, the term “Additional Benefit” in the CEE guidelines was changed to “Improvements in health outcome relative to comparator (additional benefits)” to distinguish it from the concept of usefulness in official drug pricing. This change reflects the recognition that “usefulness” in official drug pricing and “usefulness” in CEE are distinct, and suggests that consideration should be given going forward to how these concepts, while different, can be better aligned [21].

The policy implications of these findings are as follows. First, as cost-effectiveness evaluation constitutes a component of the drug pricing system, ensuring consistency with the premiums granted during the initial drug pricing process is essential. Therefore, HTA considerations should be incorporated from the pre-approval stage through the initial drug pricing process. Second, greater flexibility in value assessment could be achieved by either adjusting the interpretation of CEE results to account for premium components that are difficult to capture using HRQoL measures or excluding such components from the evaluation scope. Third, the timing of CEE should be carefully considered to allow sufficient accumulation of clinical and real-world data. In particular, conducting CEE after relevant data have matured may improve the validity of comparator selection and the accuracy of CEE, thereby reducing discrepancies between official drug pricing and CEE.

This study has several limitations. First, the sample size was small, limiting the analysis to descriptive statistics. Second, the analysis was conducted at the product level rather than the patient-population level, which may not fully capture variation across indications and target populations. Further studies with larger sample sizes are warranted to confirm the findings.

## 5. Conclusions

This study identified several key differences between the official drug pricing system and the CEE system in Japan, particularly regarding the selection of appropriate comparators and the evaluation of additional values. Although both systems have inherent methodological limitations, reducing these differences could improve the predictability of drug prices and enhance the alignment between official drug pricing and CEE.

Compared with other countries, Japan’s CEE system is relatively new and continues to evolve. Consistent implementation of official drug pricing and CEE is necessary because minimizing the differences between the two systems can enhance the predictability of official pricing for manufacturers developing products in Japan and in countries that reference Japanese prices. Aligning CEE with the official drug pricing system is essential to ensure that innovations are appropriately valued and that pricing decisions remain transparent, predictable, and consistent with real-world evidence.

Further studies using a larger sample size, including future cases as they become available, are warranted to examine uncertainty and other factors that may influence the findings.

## Figures and Tables

**Table 1:** Characteristics of CEE systems [8,9,10].

Country	Japan	United Kingdom	France	Germany
Overview of healthcare insurance system	Universal coverage through a social health insurance system	Universal coverage through the National Health Service	Universal coverage through public sickness insurance and private insurance	Universal coverage through public and private health insurance
Representative HTA agency	Center for Outcomes Research and Economic Evaluation for Health (C2H)	NICE	HAS	IQWiG
Year of implementation	2019	1999	2013	2011
Primary purpose	Price adjustment	Reimbursement decisions	Price negotiations	Price negotiations
Products subject to evaluation	Products with substantial financial impact, etc.	Products selected by the government	Products with substantial financial impact	Almost all new drugs

**Table 2:** Official drug pricing and CEE.

	Official Drug Pricing	CEE
Scope	All prescribed drugs	Selected products based on the criteria
Decision criteria	Efficacy, safety and incentive	ICER and QALY
Objectives	Pricing for broad and rapid reimbursement	Adjust the drug price based on the ICER
Evaluation process	Drug price is determined based on the pricing rule by MHLW within 60–90 days after regulatory approval	Company analysis (9 months) after selected for HTA, after company analysis public group re-analysis (9 months) + Chuikyo decision
Expected outcomes	Initial price is determined for reimbursement	Price adjustment

**Table 3:** Summary of the Product Narrowing Process.

	Number of Products
Process completed (drug)	44
excluding H5	35
excluding medical device	32
excluding no premium granted	23

**Table 4:** Type of other premiums and price reduction after CEE.

Type of Premium	Number of Products	
	No Price Reduction After CEE	Price Reduction After CEE
New MOA	1	6
Efficacy for insufficient efficacy cases	3	7
Standard treatment	1	5
Convenience	1	4
Reduction in invasiveness	1	1

**Table 5:** Difference in comparators between official drug pricing and CEE (by price adjustment category).

Number of Products	Total	No Price Reduction After CEE	Price Reduction After CEE
Same comparator	3	0	3
Different comparator: Non-drug	4	0	4
Different comparator: New drug	2	2	0
Different comparator: Old drug	14	4	10

## Data Availability

The data supporting the findings of this study are derived from publicly available reports published by the Center for Outcomes Research and Economic Evaluation for Health (C2H) and the Central Social Insurance Medical Council (Chuikyo). These data are accessible via URLs provided in the references. The curated dataset of 23 products generated during this study is available from the corresponding author upon reasonable request.

## Abbreviations

The following abbreviations are used in this manuscript:

CEE	cost-effectiveness evaluation
HRQoL	health-related quality of life
HTA	health technology assessment
QALY	quality adjusted life years
QoL	quality of life

## Appendix A

**Table A1:** Factors for innovativeness/usefulness premium [6,7].

Factors	Subdivided Factors for Innovativeness/Usefulness Premium		Points
(i) A novel, clinically useful mechanism of action (Calculated as the sum of the points for the applicable items (either a or b, but not both))		(a) Mechanism of action is markedly different from the existing drugs	2
		(b) Mechanism of action is different from the existing drugs	1
		(c) (only if (a) or (b) is fulfilled) The drug is indicated for a serious disease with no standard treatment	+1
		(d) (only if (a) or (b) is fulfilled) Clinical usefulness is demonstrated based on drug discovery and manufacturing processes that are markedly different from those of existing similar drugs	+1
		(e) (only if (a) or (b) is fulfilled) No new drug with a novel mechanism of action has been listed for a prolonged period in the same disease area	+1
		(f) (only if (a) or (b) is fulfilled) DPO recognizes the mechanism of action as clinically extremely useful	+1
(ii) Evidence of higher efficacy or safety compared to the existing similar drugs (the total points are calculated by multiplying the points in (ii)-1 by the points in (ii)-2)	(ii)-1 Higher efficacy or safety (the sum of the points for the applicable items)	(a) The drug shows higher efficacy in a clinically important efficacy endpoint	1
		(b) This drug shows higher safety in a clinically important safety endpoint	1
		(c) (only if (a) or (b) is fulfilled) DPO recognizes the efficacy and/or safety as clinically extremely useful	+1
	(ii)-2 Evidence level (Either one (but not both))	(a) Supported by randomized clinical trials	2
		(b) Supported by other methods with objectivity and reliability, such as where, for new drugs targeting intractable or rare diseases for which comparative trials are difficult to conduct due to factors including small patient populations, higher efficacy and/or safety compared to existing similar drugs is objectively and reliably demonstrated based on results from single-arm studies and is recognized by the drug pricing organization	1
(iii) Evidence of improvement treatment in the treatment method of the target disease (the sum of the points for the applicable items)		(a) The drug shows efficacy in patients resistant/intolerant to the existing therapies	1
		(b) The drug is recommended as a standard treatment	1
		(c) The drug shows efficacy in a more speedy or long-lasting manner	1
		(d) The drug shows a clinically important combination effect with existing therapies	1
		(e) The drug shows high efficacy in a specific patient population defined based on the mechanism of action	1
		(f) The drug shows improvement in clinically important secondary endpoints, such as patient quality of life (QoL), compared to existing treatment methods	1
		(g) DPO recognizes the improvement as clinically extremely important	1
		(h) (only if one of (a)–(e) is fulfilled) The drug is indicated for a serious disease with no standard treatment	+1
(iv) Evidence of higher clinical usefulness due to formulation improvements compared to the existing similar drugs (the sum of the points for the applicable items)		(a) The drug reduces invasiveness	1
		(b) The drug improves convenience in administration	1
		(c) The drug has a stable blood concentration	1
		(d) DPO recognizes other remarkable clinical usefulness	1

Note: One point is assigned when a novel combination product demonstrates higher efficacy compared to its individual components. Note: Reduction rule: If the Drug Pricing Organization determines that a reduction in the premium rate is particularly necessary—such as when the magnitude or scope of efficacy demonstrated in clinical trials is limited—one point may be deducted from the total points calculated according to (i–iv) above.

**Table A2:** CEE selection criteria [5].

Classification		Selection Criteria
Newly listed products * (meaning listed on health insurance after formal implementation)	H1 **	Estimated peak annual sales are ¥10 billion or more
	H2 ** ***	Estimated peak annual sales are between ¥5 billion and ¥10 billion
	H3	Products with notably high prices **** Products requiring re-evaluation because robust new evidence with a major effect on evaluation has been discovered after completion of cost-effectiveness evaluation
Already listed products (meaning listed before formal implementation) *****	H4	Products with annual sales of ¥100 billion or greater Products with notably high prices **** Products requiring re-evaluation because robust new evidence with a major effect on evaluation has been discovered after the completion of cost-effectiveness evaluation
Similar products	H5	Products whose prices are calculated comparatively against those categorized in the H1 to H4 classifications

* Products with premiums in the similar efficacy (category) comparison method, or products with premiums or disclosure rates of 50% in the cost-accounting method are prerequisite conditions for the targets of scope in the cost-effectiveness evaluation. In addition, if these products meet any of the following criteria, they are selected as target products, and are classified into 3 categories: (1) estimated peak annual sales of ¥10 billion or more (“H1 classification”), (2) estimated peak annual sales of between ¥5 billion and ¥10 billion (“H2 classification”), and (3) products requiring re-evaluation or products with notably high unit prices (“H3 classification”). ** Even if a product does not meet the selection criteria in terms of estimated peak sales at the time of listing, it will be sorted as falling into a particular classification if the annual market size exceeds the criteria due to market expansion. In this case, the product will be sorted into the H1 or H2 classifications according to their annual market size. *** Products of H2 classification are initially chosen as candidate products for evaluation; they are subsequently selected as targets. **** Notably high price is not defined explicitly, but at least a product whose unit price is JPY a few million or higher is considered to meet this criterion. ***** Regardless of the pricing methods, products with annual sales of ¥100 billion or more owing to market expansion, or products with notably high unit prices, are selected as the scope of target in the CEE, provided that the products have premiums (“H4 classification”).

**Table A3:** Product information.

Number	C2H ID	Product Name	Generic Name	ATC	Market Estimation (Peak Estimate, Unit: JPY 100 Million)	Official Drug Pricing						Price Revision After CEE	HTA Category
						Pricing Method	Comparator in Drug Pricing	Usefulness Premium	Innovativeness/Usefulness Premium Items Recognized in Price Determination	Other Premium	Applied Premium Rate		
1	C2H1901	Trelegy (Trelegy 100 Ellipta 14 doses)	Fluticasone, Umeclidinium, Vilanterol	R03AL08	236	Similar efficacy comparizon method (I)	ANORO ELLIPTA 30doses	10%	(iii) Improvement treatment in the treatment method of the target disease: ③ − a = 1p, ③ − c = 1p	NA	10%	99.5%	H1
2	C2H1902	Kymriah	Tisagenlecleucel	L01XX71	72	Cost accounting method	NA	35%	(i) Novel Mechanism of action: ① − b = 1p (iii) Improvement treatment in the treatment method of the target disease: ③ − a = 1p 5p (Utility I) + 1p + 1p = 7p	Marketability premium: 10%	9%	95.7%	H3
3	C2H1903	Ultomiris	Ravulizumab	L04AA43	331	Similar efficacy comparizon method (I)	SOLIRIS for Intravenous Infusion 300mg	5%	(iii) Improvement treatment in the treatment method of the target disease: ③ − c = 1p	NA	5%	95.7%	H1
4	C2H1905	Trintellix	Vortioxetine	N06AX26	236	Similar efficacy comparizon method (I)	LEXAPRO Tablets 10mg	5%	(i) Novel Mechanism of action: ① − b = 1p	NA	5%	95.7%	H1
5	C2H1906	Coralan	Ivabradine	C01EB17	57.5	Similar efficacy comparizon method (I)	Acardi Capsules 1.25	35%	(i) Novel Mechanism of action: ① − b = 1p (iii) Improvement treatment in the treatment method of the target disease: ③ − a = 1p	NA	35%	100.0%	H2
6	C2H2002	Cabometyx	Cabozantinib	L01EX07	127	Similar efficacy comparizon method (I)	SUTENT Capsule 12.5mg	10%	(ii) Higher efficacy or safety compared to the existing similar drugs (incl. evidence level): ② − 1 − a, ② − 2 − a = 1p × 2 = 2p	NA	10%	100.0%	H1
7	C2H2003	Enhertu	Trastuzumab Deruxtecan (Genetical Recombination)	L01XC41	129	Similar efficacy comparizon method (I)	KADCYLA for Intravenous Infusion	5%	(iii) Improvement treatment in the treatment method of the target disease: ③ − a = 1p	NA	5%	97.8%	H1
8	C2H2007	Rybelsus	Semaglutide (Genetical Recombination)	A10BJ06	116	Similar efficacy comparizon method (I)	Ozempic Subcutaneous Injection 2mg SD	5%	(iv) Higher usefulness due to formulation improvements compared to the existing similar drugs: ④ − a = 1p	NA	5%	97.5%	H1
9	C2H2103	Polivy	Polatuzumab Vedotin	L01XC37	120	Similar efficacy comparizon method (I)	ADCetris for I.V. Infusion 50mg	5%	(iii) Improvement treatment in the treatment method of the target disease: ③ − b = 1p	NA	5%	100.0%	H1
10	C2H2104	Darzquro	Daratumumab Vorhyaluronidase Alfa	L01FC01	370	Similar efficacy comparizon method (I)	DARZALEX Intravenous Infusion	5%	(iii) Improvement treatment in the treatment method of the target disease:③ − c = 1p	NA	5%	100.0%	H1
11	C2H2105	Arikayce	Amikacin sulfate	D06AX12 J01GB06 S01AA21	177	Cost accounting method	NA	10%	(iii) Improvement treatment in the treatment method of the target disease:③ − a, b = 2p	NA	2%	90.6%	H1
12	C2H2110	Revestive	Teduglutide (Genetical Recombination)	A16AX08	60	Cost accounting method	NA	5%	(iii) Improvement treatment in the treatment method of the target disease: ③ − a = 1p	Marketability premium: 10%	3%	92.9%	H2
13	C2H2113	Retevmo	Selpercatinib	L01EX22	156	Similar efficacy comparizon method (I)	XALKORI Capsules	5%	(i) Novel Mechanism of action: ① − b = 1p	Marketability premium: 10%	15%	99.8%	H1
14	C2H2114	Padcev	Enfortumab vedotin	L01FX13	118	Similar efficacy comparizon method (I)	KADCYLA for Intravenous Infusion	10%	(iii) Improvement treatment in the treatment method of the target disease: ③ − a, ③ − b = 2p	NA	10%	91.8%	H1
15	C2H2203	Bimzelx	Bimekizumab (Genetical Recombination)	L04AC21	120	Similar efficacy comparizon method (I)	Cosentyx for s.c. injection syringe	5%	(ii) Higher efficacy or safety compared to the existing similar drugs (incl. evidence level): ② − 1 − a, ② − 2 − b = 1p	NA	5%	100.0%	H1
16	C2H2205	Dysval	Valbenazine Tosilate	N07XX13	62	Similar efficacy comparizon method (I)	CHOREAZINE Tablets 12.5mg	5%	(iii) Improvement treatment in the treatment method of the target disease: ③ − b = 1p	NA	5%	98.6%	H2
17	C2H2208	Lagevrio	Molnupiravir	J05AB18	138	Similar efficacy comparizon method (I)	VEKLURY	10%	(iii) Improvement treatment in the treatment method of the target disease: ③ − b/c = 2p	NA	10%	91.8%	H1
18	C2H2209	Sotyktu	Deucravacitinib	L04AF07	225	Similar efficacy comparizon method (I)	Otezla Tablets	40%	(i) Novel Mechanism of action: ① − b = p (ii) Higher efficacy or safety compared to the existing similar drugs (incl. evidence level): ② − 1 − a, ② − 2 − a = 2p	NA	40%	91.4%	H1
19	C2H2210	Tezspire	Tezepelumab (Genetical Recombination)	R03DX11	145	Similar efficacy comparizon method (I)	Nucala solution for s.c. injection	5%	(i) Novel Mechanism of action: ① − b = 1p	Pediatric premium: 5%	10%	95.9%	H1
20	C2H2212	Mounjaro	Tirzepatide	A10BX16	367	Similar efficacy comparizon method (I)	Ozempic Subcutaneous Injection 2mg SD	10%	(ii) Higher efficacy or safety compared to the existing similar drugs (incl. evidence level): ② − 1 − a, ② − 2 − a = 2p	NA	10%	100.0%	H1
21	C2H2213	XOCOVA	Ensitrelvir Fumaric Acid	J05AE16	192	Calculated based on the special rule applicable to this product.	LAGEVRIO Capsules 200mg/XOFLUZA Tablets 20mg	5%	(i) Novel Mechanism of action: ① − b = 1p	NA	5%	95.7%	H1
22	C2H2303	Litfulo	Ritlecitinib tosylate	L04AF08	156	Similar efficacy comparizon method (I)	Olumiant tablets	5%	(iii) Improvement treatment in the treatment method of the target disease: ③ − a = 1p	Pediatric premium: 5%	10%	96.2%	H1
23	C2H2307	EPKINLY	Epcoritamab	L01FX27	307	Similar efficacy comparizon method (I)	BLINCYTO For I.V. Infusion 35 μg	10%	(iii) Improvement treatment in the treatment method of the target disease: ③ − b, ③ − c = 2p	NA	10%	97.3%	H1
